# Changes in Families’ Leisure, Educational/Work and Social Screen Time Behaviours before and during COVID-19 in Australia: Findings from the Our Life at Home Study

**DOI:** 10.3390/ijerph182111335

**Published:** 2021-10-28

**Authors:** Lauren Arundell, Jenny Veitch, Shannon Sahlqvist, Riaz Uddin, Nicola D. Ridgers, Jo Salmon, Anna Timperio, Kate Parker

**Affiliations:** Institute for Physical Activity and Nutrition (IPAN), School of Exercise and Nutrition Sciences, Deakin University, Geelong 3220, Australia; jenny.veitch@deakin.edu.au (J.V.); shannon.sahlqvist@deakin.edu.au (S.S.); r.uddin@deakin.edu.au (R.U.); nicky.ridgers@deakin.edu.au (N.D.R.); jo.salmon@deakin.edu.au (J.S.); anna.timperio@deakin.edu.au (A.T.); k.parker@deakin.edu.au (K.P.)

**Keywords:** child, adolescent, family, television, leisure activities, social interaction

## Abstract

This study aimed to understand differences in leisure, educational/work and social screen time behaviours experienced by parents and children due to COVID-19 lockdown restrictions, which may inform behaviour change strategies and policy in the transition to a COVID-normal life. Participants in the “Our Life at Home” study (*n* = 218 parents from Australia, 43.4 ± 6.8 years, 88% female) completed a cross-sectional online survey in April/May 2020. Parents recalled their own and their child (8.7 ± 2.0 years, 42% female) or adolescents (15.0 ± 1.5 years, 50% female) participation in nine screen time behaviours in the past month (during lockdown) and retrospectively for February 2020 (pre-lockdown), providing data on 436 individuals. Screen time behaviours included leisure (computer/laptop and tablet/smartphone for leisure, TV/videos/DVDs and game consoles); education/work (computer/laptop and tablet/smartphone for work/education); and social screen time (computer/tablet/smartphone for social communication with friends, family and work (parents only)). Wilcoxon signed-rank tests and effect sizes (*r*) compared the time spent in each behaviour pre-lockdown and during lockdown. Large differences were observed in social (parents: *r* = 0.41–0.57; children: *r* = 0.55–0.65; adolescents: *r* = 0.28–0.43) and education (children: *r* = 0.50–0.65 and adolescents: *r* = 0.25–0.37) behaviours. There were small or no differences in leisure time screen use. COVID-19 lockdown restrictions have impacted parent’s and children’s screen time, and future research and policy should consider strategies to support families to manage screen time.

## 1. Introduction

Excessive screen time (i.e., the use of electronic devices, such as smartphones, digital tablets, computers and televisions (TV)), particularly during leisure time, places children and adults at increased risk of poor physical and psychosocial health. For example, excessive screen time in childhood can increase the risk of obesity and cardio-metabolic disease risk factors [1,2], myopia [3], poor mental health [4], lower social skills [5], lower school attainment [6] and lower social connectedness [7]. Similar associations are noted amongst adults [8,9,10]. Further, there is growing evidence that some of these associations are screen behaviour specific [6,7,11]. Evidence shows positive associations between educational screen use and academic benefits and negative associations between social screen use, quality of life and socio-emotional health in youth [6]. Consistent with other countries [12], Australian recommendations specify that children (aged 5–17 years) should limit daily recreational/leisure screen use to no more than 2 h per day, yet almost two-thirds (65%) of Australian children aged 5–12 years do not meet this guideline, and adherence declines further with increasing age [13]. Whilst there are currently no screen time recommendations for Australian adults, evidence shows parents and children often display similar screen behaviours [14], which may be amplified when family members spend more time at home, as occurred during COVID-19 lockdown restrictions.

The COVID-19 pandemic resulted in government-enforced lockdown restrictions worldwide. Within Australia, nation-wide restrictions, including the closure of schools and workplaces, meant that families experienced unprecedented movement restriction and social isolation and were generally required to work and study from home. These changed circumstances likely impacted engagement in screen behaviours; however, little research has examined changes in Australians’ screen time behaviours during COVID-19 lockdown restrictions. Amongst the studies from other countries experiencing COVID-19 lockdown restrictions that have shown that children’s and adults’ screen time changed, the screen time behaviour assessment has typically centred on guideline adherence, single types of screen use or combined ‘screen time’ despite behaviour-specific health outcomes. For example, only 11% of Canadian children (5–17 years) met screen time guidelines during their lockdown [15], and adults (18+ years) in the USA recalled engaging in greater overall total screen time (assessed as average daily screen time) compared to pre-lockdown restrictions [16]. A study of youth (4–17 years) from Germany found that children and adolescents engaged in more leisure screen time activities (assessed as combined TV viewing, gaming and recreational internet use) during their lockdown, and the magnitude of change was greatest amongst adolescents compared to children [17]. Less is known about the impact of COVID-19 lockdown restrictions on Australian children’s screen time. Only one study examined screen time behaviours of 5–9-year-old children from Western Australia and reported that their weekly leisure screen time (assessed as combined TV, DVD, video, computer, smartphone and game console use) doubled under lockdown restrictions [18]. Critically, lockdown restrictions resulted in unprecedented changes in the ways in which families and individuals engaged with screens, whether to work, to learn, to connect or to relax, and, as such, it is important to capture changes in a wide variety of screen behaviours. In particular, recent evidence has shown that prior to lockdowns, children and adults engage in a variety of screen time behaviours [19], and this is likely to have changed further as a result of the COVID-19 lockdown restrictions. Understanding the changes in a wide range of screen time behaviours, including leisure, educational/work and social screen use, is important to inform strategies and policy to promote optimal screen use as restrictions ease and ‘COVID-19 normal’ is pursued. Therefore, the primary aim of this study was to examine differences in the duration of leisure, educational/work and social screen time behaviours from pre-COVID-19 restrictions (February 2020) to lockdown restrictions (April/May 2020) among parents and children in Australia. A secondary aim was to examine differences within child (5- < 12 years) and adolescent (12- < 18 years) age groups.

## 2. Materials and Methods

In Australia, “stay-at-home” lockdown restrictions (hereon referred to as “lockdown restrictions”) were introduced on 23rd March 2020 by the Federal Government and began easing 1st May 2020. During this time, the only four permitted reasons to leave home were for exercise, shopping for essentials, caring for self and others and work/study that could not be done at home. The restrictions in place across the states and territories of Australia during this study’s recall period are described elsewhere [20].

Data were drawn from the “Our Life at Home” (OL@H) study for analysis. OL@H is a purpose-designed longitudinal study aiming to understand the impact of the COVID-19 pandemic on Australians’ physical activity, sedentary behaviour, health and wellbeing. This paper uses cross-sectional data collected in April/May 2020, with retrospective recall of behaviours in February 2020. This study received approval from the Deakin University Human Ethics Advisory Group-Health (HEAG-H 59_2020).

Methods have been described previously [20]. In brief, consenting participants (*n* = 6474, aged 13–75 years) completed an online survey in April/May 2020, self-reporting their own behaviours. Of these, 4079 were adolescents (aged 13- < 18 years) and 2395 were adults (aged 18+ years). Adult participants were asked if they had a child aged 5–17 years, and those who indicated yes (*n* = 228) were invited to proxy-report behaviours for their child. This sub-group is the focus of the current study. Within the survey, participants provided demographic information (age, sex), employment details (days and hours working at their physical work location per week in the last week in February and April/May) and child schooling details (days attended physical school per week in the last week in April/May). In addition, participants were asked to recall their own and their child’s participation (yes/no) and duration (minutes on weekdays and weekend days during a usual week) in nine screen time behaviours during February 2020 (indicative of behaviours pre-COVID-19 lockdown) and during the past month (April/May 2020, indicative of behaviours during lockdown).

The screen time behaviour survey items used build on research by the authors, including a systematic review of the prevalence of children’s after school sedentary behaviours [21] and a purpose-designed study to identify the types of sedentary screen behaviours families perform [19]. The survey items were adapted from survey measures that members of the research team previously developed to assess movement behaviours of physical activity, sedentary behaviour and screen time behaviours and which have shown acceptable reliability (ICC 0.41–0.91) [14,22] and convergent validity compared to the same child-report survey items (ρ of 0.44–0.61 [23]). The screen time behaviours examined included (1) leisure screen time behaviours (computer/laptop for leisure, e.g., to play games, stream movies; TV/videos/DVDs; tablet/smartphone use for leisure, e.g., scrolling social media, playing games; and game consoles (e.g., Xbox^®^/Nintendo Switch^®^)); (2) educational or work screen time behaviours (computer/laptop for work/education as prescribed by school or workplace; tablet/smartphone for work/education as prescribed by school or workplace); and (3) social screen time behaviours (computer/tablet/smartphone for social communication with friends, family and for parents only for social communication for work, e.g., Zoom, FaceTime, Skype). Average duration/day in each screen time behaviour was calculated as [(weekday minutes × 5) + (weekend minutes × 2)]/7. Where a participant indicated that they did not perform an activity, that variable was coded as zero (0). As data were non-normally distributed (positively skewed), Wilcoxon signed-rank tests were used to compare the time (median minutes per day and interquartile range (IQR)) that parents and all children (aged 5- < 18 years) spent in each of the screen time behaviours between February 2020 and April/May 2020. Additional sub-group analysis by child age was conducted to explore screen time differences within child (5- < 12 years) and adolescent (12- < 18 years) age groups. Effect sizes (*r*) were calculated (z/square root of *n* [24]). Values approximating 0.10, 0.30 and ≥0.50 represent small, moderate and large effect sizes, respectively [25].

## 3. Results

In total, 218 participants reported having a child aged 5- < 18 years old provided the required data to be included in the analyses (i.e., parental self-report and child proxy-report data). Of these parents, 130 had a child aged 5- < 12 years and 88 had an adolescent aged 12- < 18 years. Participant characteristics are shown in Table 1.

Table 2 shows the median daily duration of time that parents and all children (aged 5- < 18 years) spent engaged in screen time for leisure, education/work and social connection in pre-lockdown and during lockdown.

### 3.1. Parent’s Screen Time Behaviours

Wilcoxon signed-rank tests comparing parents’ time spent in screen time behaviours pre-lockdown to during lockdown found a statistically significant difference in three leisure screen time behaviours (i.e., watching TV/videos/DVDs, using tablet/smartphone for leisure and using game consoles), one education/work screen time behaviour (i.e., computer/laptop for work/education) and all three social screen time behaviours (i.e., computer/tablet/smartphone for communication with friends, family and work) (Table 2). There were large effect sizes for the differences in all social screen time behaviours (*r* ranged from 0.41–0.57) (Table 2).

### 3.2. Children’s Screen Time Behaviours

Table 2 shows that there were statistically significant differences in the amount of time that children spent pre-lockdown compared to during lockdown in one leisure screen time behaviour (tablet/smartphone for leisure), both education screen time behaviours (computer/laptop and tablet/smartphone) and both social screen time behaviours (computer/tablet/smartphone for communication with friends and family). There were large effect sizes for the differences in time spent in education (*r* ranged from 0.41–0.54) and social screen time behaviours (*r* ranged from 0.44–0.55) (Table 2).

Differences within child (5- < 12 years) and adolescent (12- < 18 years) age groups are shown in Table 3. There was a significant difference in the time that children spent using tablets/smartphones for leisure (*r* = 0.31) during lockdown compared to pre-lockdown. There were no other significant differences in leisure screen time behaviours. Children and adolescents spent more time engaged in all screen time behaviours for education/work and social connections during lockdown compared to pre-lockdown. The effect size showed that these differences were moderate to large, and the magnitude of difference was greater amongst children (*r* ranging from 0.50 to 0.68) than adolescents (*r* ranging from 0.25 to 0.43).

## 4. Discussion

This study identified the differences in leisure, education/work and social screen time behaviours that parents and children performed from pre-COVID to during the COVID-19 lockdown restrictions in Australia in 2020. Based on retrospective parent reports, parents and children spent more time engaged in almost all screen time behaviours during lockdown compared to pre-lockdown. The extent of the differences can be highlighted by extrapolating the current findings (median minutes/day) to weekly differences. During lockdown restrictions, parents performed an additional 14 h and 39 min of screen time per week, and children performed an additional 26 h and 49 min of screen time per week during lockdown compared to pre-lockdown (excluding any unmeasured screen multi-tasking). To date, most evidence has focused only on leisure time screen use during lockdown restrictions (e.g., TV viewing) [17,18]; however, within this study, the greatest differences reported amongst parents, all children, and children and adolescents when assessed separately were in social and education/work-based screen time behaviours. As such, this study adds to the growing global evidence showing screen time increased during lockdown restrictions [15,17,18] and provides new insights into a broader range of specific screen time behaviours among parents and their children. A recent (2021) meta-analysis of age-related changes in children’s (5–18 years) sedentary behaviour showed that over 1 year, children typically accumulate an additional 21 min of screen time per day [26]. The lockdown measures may have exacerbated this expected rate of increase whereby increases in screen time were equivalent to those typically observed over many years. While the observed changes in screen use were driven by an immediate, unprecedented and temporary change in lifestyle, they may result in a permanent change in screen time exposure, participation and expectations, leading to long-term impacts on psychosocial [27,28] and physical health [3]. These findings should therefore underpin the prioritization, development and adaptation of strategies targeting parents’, children’s and adolescents’ screen time to ensure that they address the needs of families within the changed screen time environment.

It is important for families, communities, and workplaces to support and promote face-to-face interactions in the wake of the COVID-19 lockdown restrictions to reduce dependence on screen use. The largest differences between pre-lockdown and during lockdown restrictions in the current study were reported in screen time for social connection with friends, with family and for work (parents only). Screen time has been shown to be negatively associated with social development [5], social quality of life [6] and social connectedness in youth [7] and adults [10]. However, COVID-19 lockdown restrictions resulted in screens being one of the only methods for people to communicate with others outside their house, and the desire to connect with others has been noted as a driver for this change in screen time [29]. Importantly, the magnitude of differences in screen use for social communication between pre-lockdown and during lockdown restrictions was larger amongst children compared to adolescents. This suggests that the lockdown restrictions may have resulted in children being introduced to and using screens for social communication earlier than they would have otherwise. Australian [30] and international [31] evidence shows that screen time increases during childhood and adolescence, and early exposure may exacerbate this trajectory; therefore, continued research to inform policy and practice is warranted [32].

The requirement for remote schooling and work resulted in the use of screens for educational and work purposes to be much greater during lockdown than pre-lockdown. The magnitude of difference in the use of computer/laptop and tablet/smartphones for educational/work purposes was larger amongst children than parents, which may be indicative of parents’ prior use of these screens for work, whereas this requirement was more recent for primary school children. Although not assessed, the higher screen use for educational purposes noted in the current study may also be attributed to parents being more aware of their child’s screen use for schooling when children were at home. As lockdown restrictions ease, it may be particularly important for schools to promote non-screen tasks for homework to reduce the reliance on the use of screens at home, as it has been shown that children who are required to use screens for homework are more likely to be characterised as high computer users in their leisure time [14]. Implementing school/teacher-delivered programs that target behaviours within the school and home settings, such as the Transform-Us! Program [33], and ensuring that before and after-school care services or programs follow screen time guidelines [34] may assist in reducing screen use in both settings.

The small or lack of difference in time spent in leisure time screen behaviours, particularly game console use and watching TV/videos/DVDs, in this study may be due to the flexible and voluntary nature of these behaviours. Previous research has shown parental rules and restrictions are associated with children’s screen time [35], and this is also evident during lockdown restrictions [15,36]. During lockdown, parents may feel more willing to enforce restrictions on leisure time screen use than restrict screen use for education or social connections. It is also possible that in previous studies, screen use for social interaction (e.g., communicating via tablets/smartphones) was captured within leisure time screen use (e.g., tablet/smartphone use) and that social interactions are obtained from leisure time screen behaviours (e.g., computer or console gaming use increasingly involves social interaction through multi-player platforms). This study shows how important it is to ensure screen time measures are constantly updated to capture the device used and behaviour performed as the technology environment changes. Interestingly, tablet/smartphone use for leisure (separate to tablet/smartphone use for social communication) became the most used leisure screen time behaviour for parents and adolescents during lockdown. The high use of tablet/smartphone for leisure amongst youth and parents has previously been identified [19], and it has further been identified as the top behaviour parents believe they and their child could reduce [19]. This finding, coupled with the small differences in TV viewing from pre-lockdown to during lockdown restrictions, reinforces the need to review screen time recommendations and intervention strategies, which have previously been established primarily based upon TV viewing literature [37,38]. For example, screen time intervention strategies that have been effective prior to lockdown restrictions (typically targeting total screen time or TV viewing time) have incorporated family involvement, parental support and role modelling, which have resulted in reduced screen time among children and parents [35,39,40]. The current findings highlight that the behaviour targets of intervention strategies need to be updated. Future research and guidelines need to focus on understanding and managing the diverse screen time devices available and the diverse behaviours parents and children currently perform [19] and determine if adapting current strategies is effective at managing screen use.

The strengths of this study include the range of screen time behaviours assessed, which captured leisure time, educational/work and social screen time behaviours, and many behaviours that have previously been overlooked, particularly in the COVID-19 context. The inclusion of data on the use of screens among parents and children as well as behaviours from before and during Australia’s COVID-19 lockdown also provide novel findings. Limitations include the relatively small sample size (although the self- and proxy-report data provide information on 436 individuals), predominantly female parents, the use of cross-sectional self- and proxy-report data, the inability to report time spent multi-tasking on multiple screens simultaneously and the length of the recall time period. However, behaviour recall is necessary and common amongst COVID-19-related studies due to the unexpected nature of the pandemic [16,18]. Further, there may be other screen time behaviours that parents and children performed that were not captured in the current study, and parents may not have been aware of all the screen time behaviours their child was performing, particularly prior to lockdown restrictions. While it was important to capture the variety of different screen behaviours that children and adults engage in due to their associations with different health and educational outcomes [6,11], it may have been difficult for participants to distinguish between these behaviours, possibly leading to over reporting. There is no reason to believe that this over reporting would have been different in February as compared with April/May. There may also be seasonal differences in screen time between February and April/May; however, this is more evident for children’s objectively measured physical activity [41,42,43] and sedentary time [41,42,43,44] with little evidence of seasonality in subjectively measured screen-based sedentary behaviours [45]. It is now crucial for longitudinal research to investigate how screen time behaviours have changed due to COVID-19 lockdown restrictions, including how they change as lockdown restrictions ease, whether these screen behaviours remain elevated in the future once lockdown restrictions end and the long-term impact on health and wellbeing. In addition, intervention strategies need to be developed in line with this comprehensive understanding of how families engage with screens for leisure, education/work and social connections.

## 5. Conclusions

This study found large differences in the amount of time parents and children performed screen time behaviours for education/work and social connections and, to a lesser extent, for leisure during COVID-19-related lockdown restrictions in Australia compared to the pre-lockdown. The differences were greatest amongst children (5–17 years), particularly younger children (5- < 12 years). Future research should examine whether these increases have endured beyond the lockdown and identify how to reduce these behaviours and implement strategies to avoid potential long-term adverse health and wellbeing implications. Further, there is an urgent need to develop effective strategies that support families to manage screen time after this substantial alteration.

## Figures and Tables

**Table 1:** Participant characteristics.

	Parents (*n* = 218)	All Children (5- < 18 yrs; *n* = 218)	Child (5- < 12 yrs; *n* = 130)	Adolescents (12- < 18 yrs; *n* = 88)
Age, mean years (±SD)	43.4 (±6.8)	11.2 (±3.6)	8.7 (±2.0)	15.0 (±1.5)
Female (%)	88%	45%	42%	50%
Employed full-time in February 2020, %	36%	-	-	-
Employed full-time in April/May 2020, %	27.0%	-	-	-
Hours/week at physical workplace, in February 2020, median (IQR)	26.8 (17.0, 35.0)	-	-	-
Hours/week at physical workplace, April/May 2020, median (IQR)	0 (0, 12.0)	-	-	-
Days/week attending physical school in April/May 2020, median (IQR) ^a^	-	0 (0, 1.0)	0 (0, 1.0)	0 (0, 1.0)

Note: yrs = years; ^a^ essential workers (e.g., nurses, emergency services) were able to attend work at their physical workplace and their children were able to attend school in April/May; SD = standard deviation; IQR = interquartile range.

**Table 2:** Duration (median minutes per day, IQR) that parents and children engaged in screen time behaviours pre-lockdown (February 2020) and during (April/May 2020) lockdown restrictions.

	Parents, *n* = 218					All Children, *n* = 218				
	Pre-Lockdown, Median (IQR)	During Lockdown, Median (IQR)	*z*-Value	*p*-Value	Effect Size	Pre-Lockdown, Median (IQR)	During Lockdown, Median (IQR)	*z*-Value	*p*-Value	Effect Size
Leisure										
Watching TV/videos/DVDs	120.0 (27.9, 180.0)	120.0 (44.3, 210.0)	2.920	0.004	0.20	60.0 (0, 120.0)	65.0 (0, 124.3)	0.881	0.378	0.07
Computer/laptop for leisure	0 (0, 60.0)	0 (0, 60.0)	0.816	0.415	0.06	0 (0, 85.7)	15.7 (0, 115.7)	1.772	0.076	0.13
Tablet/smartphone for leisure	120.0 (60.0, 214.3)	124.3 (0, 240.0)	2.145	0.032	0.16	38.6 (0, 98.6)	60.0 (0, 137.1)	2.749	0.006	0.21
Game consoles ^a^	-	-	-	-	-	0 (0, 34.3)	0 (0, 34.3)	2.167	0.030	0.16
Education/work										
Computer/laptop for work/education	237.9 (14.3, 384.3)	265.7 (68.6, 454.3)	7.239	<0.001	0.15	9.6 (0, 192.9)	171.4 (21.4, 265.7)	7.239	<0.001	0.41
Tablet/smartphone for work/education	1.8 (0, 85.7)	21.4 (0, 98.6)	1.761	0.078	0.12	0 (0, 0)	0 (0, 42.9)	5.534	<0.001	0.54
Social										
Computer/tablet/smartphone, communication with friends	0 (0, 30.0)	17.1 (0, 60.0)	6.930	<0.001	0.47	0 (0, 17.1)	21.4 (0, 77.1)	7.329	<0.001	0.55
Computer/tablet/smartphone, communication with family	5 (0, 30.0)	18.9 (0, 49.3)	6.028	<0.001	0.41	0 (0, 7.1)	4.5 (0, 24.3)	5.936	<0.001	0.44
Computer/tablet/smartphone, communication for work (parents only)	0 (0, 21.4)	42.9 (0, 107.0)	8.313	<0.001	0.57	-	-	-	-	-

Note: effect size calculated as (*z*/square root of *n*), and values of around 0.10, 0.30 and ≥0.50 represent small, moderate and large effect sizes, respectively. IQR = interquartile range. ^a^ median and IQR for parents were 0.0 and therefore excluded from analysis.

**Table 3:** Duration (median minutes per day, IQR) that children (5- < 12 years) and adolescents (12- < 18 years) engaged in screen time behaviours pre-lockdown (February 2020) and during (April/May 2020) lockdown restrictions.

	Children (5- < 12 years), *n* = 130					Adolescents (12- < 18 years), *n* = 88				
	Pre-Lockdown, Median (IQR)	During Lockdown, Median (IQR)	z-Value	*p*-Value	Effect Size	Pre-Lockdown, Median (IQR)	During Lockdown, Median (IQR)	z-Value	*p*-Value	Effect Size
Leisure										
Watching TV/videos/DVDs	77.1 (8.6, 120.0)	77.1 (24.3, 137.1)	1.012	0.311	0.10	60.0 (0, 120.0)	60.0 (0, 120.0)	0.160	0.872	0.02
Computer/laptop for leisure	0 (0, 34.3)	0 (0, 55.7)	1.214	0.225	0.12	77.1 (0, 188.6)	83.6 (0, 180.0)	1.267	0.205	0.15
Tablet/smartphone for leisure	0 (0, 60.0)	30.0 (0, 98.6)	3.194	0.001	0.31	77.1 (30, 154.3)	120.0 (0, 197.1)	0.664	0.507	0.08
Game consoles	0 (0, 30.0)	0 (0, 17.1)	1.334	0.182	0.12	0 (0, 68.6)	0 (0, 60.0)	1.758	0.079	0.20
Education/work										
Computer/laptop for work/education	0 (0, 30.0)	85.7 (0, 171.4)	7.006	<0.001	0.68	214.3 (21.4, 308.6)	265.7 (171.4, 394.3)	3.180	0.002	0.37
Tablet/smartphone for work/education	0 (0, 0)	0 (0, 42.9)	5.188	<0.001	0.50	0 (0, 0)	0 (0, 21.4)	2.173	0.030	0.25
Social										
Computer/tablet/smartphone, communication with friends	0 (0, 0)	12.1 (0, 41.4)	6.659	<0.001	0.65	8.6 (0, 77.1)	60.0 (17.1, 137.1)	3.732	<0.001	0.43
Computer/tablet/smartphone, communication with family	0 (0, 8.6)	8.6 (0, 30.0)	5.635	<0.001	0.55	0 (0, 1.4)	0 (0, 17.1)	2.441	0.015	0.28

Note: effect size calculated as (*z*/square root of *n*), and values of around 0.10, 0.30 and ≥0.50 represent small, moderate and large effect sizes, respectively. IQR = interquartile range.

## Data Availability

Data may be made available upon reasonable request to the authors.
